# Newborn screening for Cerebrotendinous Xanthomatosis: A retrospective biomarker study using both flow-injection and UPLC-MS/MS analysis in 20,000 newborns

**DOI:** 10.1016/j.cca.2022.12.011

**Published:** 2022-12-16

**Authors:** Frédéric M. Vaz, Youssra Jamal, Rob Barto, Michael H. Gelb, Andrea E. DeBarber, Ron A. Wevers, Marcel R. Nelen, Aad Verrips, Albert H. Bootsma, Marelle J. Bouva, Nick Kleise, Walter van der Zee, Tao He, Gajja S. Salomons, Hidde H. Huidekoper

**Affiliations:** a Amsterdam UMC Location University of Amsterdam, Departments of Clinical Chemistry and Pediatrics, Laboratory Genetic Metabolic Diseases, Emma Children’s Hospital, Meibergdreef 9, Amsterdam, the Netherlands; b Amsterdam Gastroenterology Endocrinology Metabolism, Inborn Errors of Metabolism, Amsterdam, the Netherlands; c Core Facility Metabolomics, Amsterdam UMC Location University of Amsterdam, Amsterdam, the Netherlands; d Department of Chemistry, University of Washington, Seattle, WA, USA; e University Shared Resource and Department of Chemical Physiology and Biochemistry, Oregon Health and Science University, Portland, OR, USA; f Department of Laboratory Medicine, Translational Metabolic Laboratory, Radboud University Medical Center, Nijmegen, the Netherlands; g Department of Human Genetics, Radboud University Medical Center, 6500 HB Nijmegen, the Netherlands; h Department of Neurology, Canisius Wilhelmina Hospital, Nijmegen, the Netherlands; i Center for Health Protection, National Institute for Public Health and the Environment, Bilthoven, the Netherlands; j PerkinElmer / Wallac Oy, Mustionkatu 6, 20750 Turku, Finland; k Department of Pediatrics, Center for Lysosomal and Metabolic Diseases, Erasmus MC University Medical Center, Rotterdam, the Netherlands; l United for Metabolic Diseases, the Netherlands

**Keywords:** Cerebrotendinous Xanthomatosis, Newborn screening, Tandem mass spectrometry, Metabolite ratios

## Abstract

**Background and aims:**

Cerebrotendinous Xanthomatosis (CTX) is a treatable disorder of bile acid synthesis caused by deficiency of 27-sterol hydroxylase -encoded by *CYP27A1*- leading to gastrointestinal and progressive neuropsychiatric symptoms. Biochemically, CTX is characterized by accumulation of the bile alcohol cholestanetetrol glucuronide (GlcA-tetrol) and the deficiency of tauro-chenodeoxycholic acid (*t*-CDCA) and tauro-trihydroxycholestanoic acid (*t*-THCA).

**Materials and Methods:**

To ascertain the feasibility of CTX newborn screening (NBS) we performed a study with deidentified Dutch dried blood spots using reagents and equipment that is frequently used in NBS laboratories. 20,076 deidentified newborn blood spots were subjected to flow-injection (FIA)-MS/MS and UPLC-MS/MS analysis to determine the concentration of GlcA-tetrol and calculate the GlcA-tetrol/*t*-CDCA and *t*-THCA/GlcA-tetrol ratios.

**Results:**

Using UPLC-MS/MS analysis both GlcA-tetrol concentration and/or metabolite ratios GlcA-tetrol/*t*-CDCA proved to be informative biomarkers; newborn DBS results did not overlap with those of the CTX patients. For FIA-MS/MS, GlcA-tetrol also was an excellent marker but when using the combination of the GlcA-tetrol/*t*-CDCA and *t*-THCA/GlcA-tetrol ratios also did not yield any screen positives.

**Conclusion:**

Newborn screening for CTX using only metabolite ratios following the measurement of three CTX biomarkers is possible using either FIA-MS/MS or UPLC-MS/MS, which paves the way for introduction of CTX NBS.

## 1. Introduction

Cerebrotendinous Xanthomatosis (CTX)^1^ is an autosomal recessive disorder of bile acid synthesis where the enzyme 27-sterol hydroxylase is deficient as a result of variants in the *CYP27A1* gene. Patients can present with neonatal cholestasis, bilateral cataracts, developmental delay and chronic diarrhea in childhood, and develop tendon xanthomas and various neuropsychiatric symptoms from adolescence onward [1]. Biochemically, CTX is characterized by reduced levels of the primary bile acids chenodeoxycholic acid and cholic acid, accumulation of toxic bile acid intermediates and excessive storage of cholestanol and cholesterol in tissues. As the development of symptoms can be halted/prevented by supplementation with chenodeoxycholic acid, early treatment is essential, making CTX an ideal candidate for newborn screening (NBS) [2]. In addition to the known CTX biomarker 7α,12α-dihydroxy-4-cholesten-3-one [3], we previously described two metabolite ratios for CTX, which are based on the accumulation of the cholestanetetrol glucuronide (GlcA-tetrol) and the deficiency of tauro-chenodeoxycholic acid (*t*-CDCA) and tauro-trihydroxycholestanoic acid (*t*-THCA) [4]. The two ratios, GlcA-tetrol/*t*-CDCA and *t*-THCA/GlcA-tetrol, were recently used to screen for CTX using an UPLC-MS/MS method in 32,000 deidentified newborn dried blood spots (DBS) and resulted in the identification of a genetically confirmed CTX patient without any false positives [5]. Quantifying the concentration of GlcA-tetrol, aided by the use of a stable-isotope labeled GlcA-tetrol internal standard, also was useful to screen for CTX, especially in combination with metabolite ratios [5]. To ascertain the feasibility of CTX NBS we performed a study with 20,076 deidentified Dutch DBS. We determined the GlcA-tetrol/*t*-CDCA and the *t*-THCA/GlcA-tetrol ratio as well as the GlcA-tetrol concentration comparing flow-injection analysis (FIA)-MS/MS with UPLC-MS/MS. For both methods the frequently applied Neobase^™^ 2 assay procedure was used, also to see if it can potentially be multiplexed with the existing method for analysis of amino acids and acylcarnitines.^1^

## 2. Materials and methods

### 2.1. Materials

Travere Therapeutics provided both the GlcA-tetrol and ^2^H_6_-GlcA-tetrol internal standard, which if researchers are interested in setting up the GlcA-tetrol assay can contact A. DeBarber (debarber@ohsu.edu). *t*-CDCA was from Steraloids, ^2^H_4_-*t*-CDCA was from Sigma Aldrich, *t*-THCA was prepared as described previously [6]. All solvents were MS-grade. Acetonitrile and isopropanol were from Biosolve and Methanol was obtained from Merck and LiChrosolve, formic acid was from Merck.

### 2.2. Blood spot samples

Residual heelprick blood from deidentified DBS of 20,076 newborns were obtained from the 2019 cohort of the biobank of the Dutch newborn screening program (Dutch National Institute for Public Health and the Environment, Bilthoven, The Netherlands). DBS were not used if parents objected to anonymized use of the residual heelprick blood of their child for scientific research. This is a choice of the parents that is registered at the time of collection, the standard procedure in the Dutch newborn screening program. The use of residual heelprick blood was formally approved by the national research committee for neonatal screening (WONHS) of the Dutch National Institute for Public Health and the Environment. DBS were collected from two siblings (CTX1, age 5.5 years, and CTX2, age 10 years) with genetically confirmed CTX (*CYP27A1* mutations: c.1183C > T;(p.(Arg395Cys) + c.1184 + 1G > A; (p.?)) before starting treatment with CDCA supplementation. Written informed consent was obtained prior to collection of these DBS.

### 2.3. Sample preparation, controls and eluants

3.2 mm DBS were punched in U-bottomed 96-wells plates (PerkinElmer) and 125 μl Neobase 2 extraction solution (PerkinElmer) with a final concentration of 65 nM ^2^H_6_-GlcA-tetrol and 68 nM ^2^H_4_-*t*-CDCA as internal standards was added after which the plate was sealed with an adhesive microplate cover and incubated for 30 min at 40 °C at 700 rpm using a Trinest incubator shaker (PerkinElmer). Next, 100 μl of the extract was transferred to a new 96-well plate and sealed with an adhesive microplate cover, now ready for FIA-MS/MS and UPLC-MS/MS analysis. Control samples consisted of the following blood spots: two negative controls (no additions) and two positive controls; a GlcA-tetrol/*t*-CDCA-spiked sample and a *t*-THCA-spiked sample. The two positive control DBS were prepared by diluting GlcA-tetrol/*t*-CDCA/*t*-THCA stock solution 100-fold in methanol which was added to K_2_EDTA whole blood and spotted on Eastern Business Forms paper cards. In each analytical run, DBS from CTX1 and/or CTX2 were included (CTX1 for all analytical runs, CTX2 when these blood spots became available) to ascertain the run-to-run variability and longitudinal stability of the method.

### 2.4. FIA-MS/MS and UPLC-MS/MS method

The LC system consisted of an LX-50 autosampler and pump and a QSight 220 CR mass spectrometer (PerkinElmer). Details about the protocol for the FIA- and UPLC-MS/MS method including columns, eluents, pump, autosampler and MS settings and MRM transitions can be found in supplementary material 1.

### 2.5. Data processing of MS/MS analyses

Simplicity^™^ 3Q version 1.8 was used to process both FIA- and UPLC-MS/MS data. The peak areas of GlcA-tetrol, *t*-CDCA and *t*-THCA were used to calculate GlcA-tetrol/*t*-CDCA and *t*-THCA/GlcA-tetrol ratios in Excel 2016. GlcA-tetrol was quantified using the ^2^H_6_-GlcA-tetrol internal standard assuming identical response and concentration based on the qNMR measurement. Concentrations of *t*-CDCA and *t*-THCA were calculated using the ^2^H_4_-*t*-CDCA internal standard assuming identical response. Further data analysis and generation of graphs was performed in GraphPad Prism 9.1.

### 2.6. DNA extraction from DBSs and CYP27A1 genetic analysis

DNA extraction from 3.2 mm punches was performed using an automated liquid handler NIMBUS presto (Hamilton) configured for utilizing Mag-Bind Blood chemistry (Omega Bio-tek) for nucleic acid purification. The purified DNA was quantified using a Dropsense (Trinean). Whole-exome sequencing was performed in a selection of 67 DBS, similar to previous reports with some modifications [7]. In brief, DNA samples were processed using the Human Exome kit including extended RefSeq targets (Twist Biosciences). Libraries were prepared according to the manufacturers’ protocols. All DNA samples were sheared using a Covaris R230 ultrasonicator (Covaris), subsequently followed by 2 × 150–base pair paired-end sequencing on a Novaseq 6000 instrument (Illumina). Mean sequence coverage was aimed to be > 60-fold and 99.0 % of all target bases read 20-fold or greater, sufficient for reliable variant calling. Downstream processing was performed using an automated data analysis pipeline that included BWA mapping, GATK variant calling, and custom-made annotation [8]. After that, an *in silico* CTX single gene-panel analysis was conducted.

## 3. Results

### 3.1. Performance and results of FIA- and UPLC-MS/MS assays

For both FIA- and UPLC-MS/MS, the MRM peak areas, that is without the use of added internal standards, were used to calculate the GlcA-tetrol/*t*-CDCA and *t*-THCA/GlcA-tetrol ratios. The concentrations of GlcA-tetrol, *t*-THCA, *t*-CDCA concentrations were determined using internal standards (supplementary material 2). All numerical data for both FIA-MS/MS and UPLC-MS/MS can be found in supplementary material 3. Inter-assay precision across the whole set (time span of ~ 14 month) was satisfactory, most components and even metabolite ratios had coefficients of variations below 25 % for the two CTX patients and the healthy control for both methods (supplementary material 4).

### 3.2. Screening performance of combined metabolite ratios

**Fig. 1.**
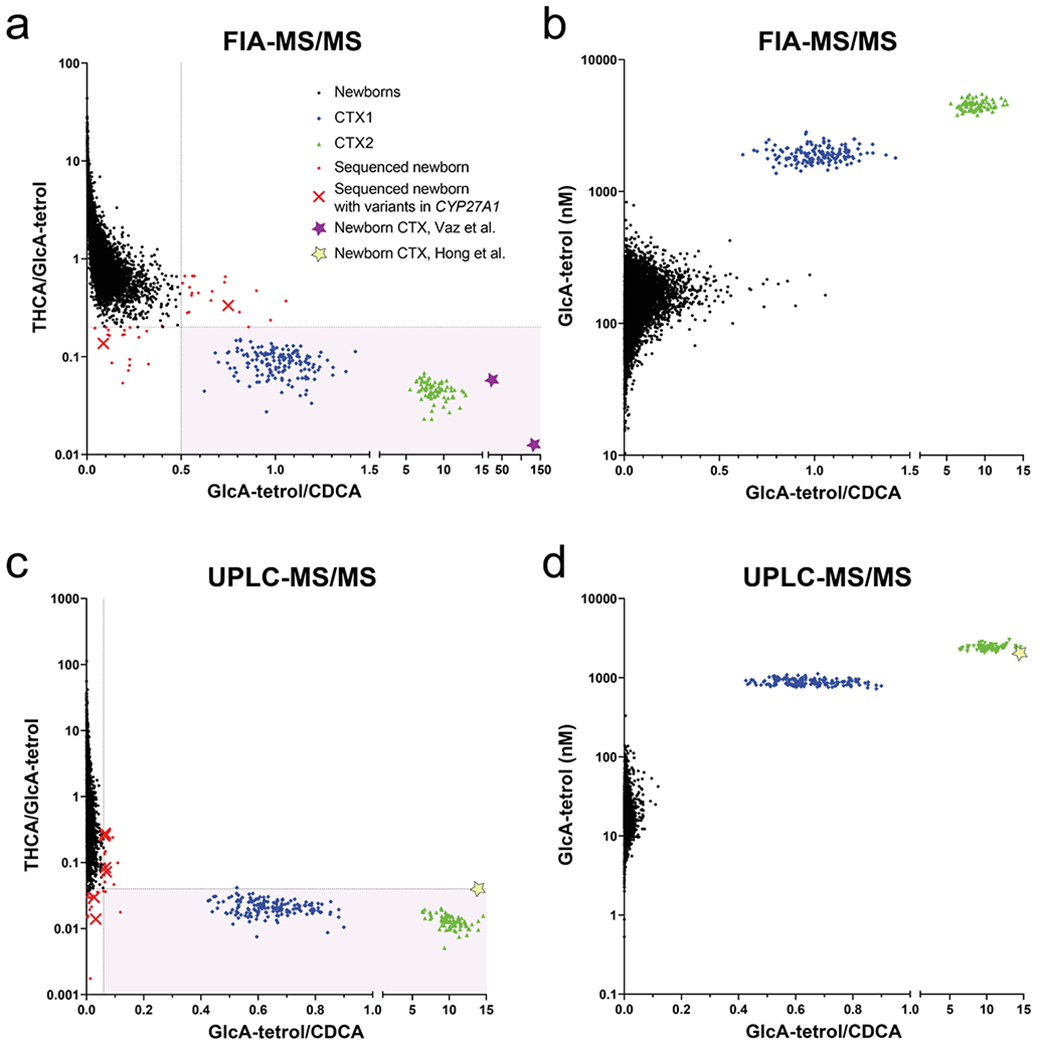
Two-dimensional representation of GlcA-tetrol/*t*-CDCA ratio vs *t*-THCA/GlcA-tetrol ratio or GlcA-tetrol concentration for both FIA-MS/MS and UPLC-MS/MS showing results for the 20,076 newborns (black dots), CTX1 (blue diamonds) and CTX2 (green triangles). CTX1 and CTX2 represent repeat samples of the same DBS collection during the 14 month of measuremnt. The pink area represents the > 99.9 % percentile for the GlA-tetrol/*t*-CDCA ratio and < 0.1 % percentile for the *t*-THCA/GlcA-tetrol ratio. Red dots are the newborns for which the *CYP27A1* gene was sequenced, red crosses are sequenced newborns where variants were found and purple and yellow stars are results of CTX newborn DBS derived from Vaz et al. [4] and Hong et al. [5], respectively.

We previously showed that the combination of both metabolite ratios or the GlcA-tetrol concentration with the GlcA-tetrol/*t*-CDCA ratio yielded a specificity and sensitivity of 100 % [5]. Fig. 1 shows plots of the GlcA-tetrol/*t*-CDCA ratio vs the *t*-THCA/GlcA-tetrol ratio or the GlcA-tetrol concentration for both FIA-MS/MS and UPLC-MS/MS of the 20,076 DBS, CTX1 and CTX2. When using either FIA- and UPLC-MS/MS, the combination of both ratios was sufficient to distinguish the newborns from CTX1 and CTX2 (Fig. 1a and 1c). Plotting the GlcA-tetrol/*t*-CDCA ratio against the GlcA-tetrol concentration showed that this distinction is considerably improved for both FIA- and UPLC-MS/MS (Fig. 1b and 1c). We also plotted results of DBS of newborn CTX individuals from two previous studies, a FIA-MS/MS method [4] (purple stars in Fig. 1a) and a UPLC-MS/MS method [5] (yellow stars in Fig. 1c and 1d), showing that these newborn CTX patients are clearly separated from newborns.

### 3.3. Further evaluation of selected DBS close to the CTX range

For both FIA-MS/MS and UPLC-MS/MS methods, we tested the DBS that had a GlcA-tetrol/*t*-CDCA ratio > 99.9 % percentile (FIA-MS/MS: >0.50; UPLC-MS/MS: >0.06) and for the *t*-THCA/GlcA-tetrol ratio those with a ratio < 0.1 % percentile (FIA-MS/MS: <0.20; UPLC-MS/MS: <0.05). This corresponded to 71 unique DBS (0.17 % of 40,152 analyses), 39 for FIA, 36 for UPLC, thus only 4 were selected in both methods. In 67 DBS sufficient DBS material was available to sequence *CYP27A1* using a whole exome sequencing approach (marked red in Fig. 1a and 1c). In three DBS a likely pathogenic variant (class 4) or a pathogenic variant (class 5) was detected without a second relevant variant (class 3 or higher), in three additional DBS only one variant of unknown significance (VUS, class 3) was present but again lacking a second relevant variant (class 3 or higher) supplementary material 5. Based on these results we did not identify CTX patients in the 20,076 deidentified newborn DBS.

## 4. Discussion

We used both FIA-MS/MS and UPLC-MS/MS to retrospectively screen 20,076 deidentified DBS of Dutch newborns for CTX to evaluate the application of metabolite ratios, GlcA-tetrol/*t*-CDCA and *t*-THCA/GlcA-tetrol, as primary screening parameters. In addition, we quantified GlcA-tetrol using a stable-isotope labeled internal standard as this has previously been shown to also be an excellent marker for CTX, especially if combined with the aforementioned metabolite ratios [5]. FIA-MS/MS yielded higher apparent levels of GlcA-tetrol and t-THCA compared to UPLC-MS/MS (supplementary material 2). This is most likely caused by the very low endogenous levels of both metabolites in non-CTX newborns and that in FIA-MS/MS the ion suppression results in a higher contribution of the background signal to the analytes leading to apparently higher concentrations in FIA-MS/MS. For the CTX patients, the GlcA-tetrol concentrations were only 2-fold higher in FIA-MS/MS corresponding to the additional peaks in the extracted ion chromatogram of the UPLC-MS/MS analysis that were not integrated but did contribute to the FIA-MS/MS GlcA-tetrol signal (supplementary material 2). This, however, did not diminish the ability to distinguish CTX1 and CTX2 from the 20,076 newborns (Fig. 1). In this study, we focused on the sole use of the two metabolite ratios, thus without the need for added internal standards, and selected newborn DBS with a GlcA-tetrol/t-CDCA ratio higher than the 99.9 % percentile and DBS with a *t*-THCA/GlcA-tetrol ratio lower than the 0.1 % percentile from both FIA- and UPLC-MS/MS analyses. Sequencing of *CYP27A1* in this selection of 67 DBS did not identify individuals with a combination of variants that would be considered to cause CTX. In six DBS, one *CYP27A1* class 3/4/5 variant was present, but all lacked a second relevant variant. None of these newborns, however, had both ratios in the respective > 99.9 % (GlcA-tetrol/*t*-CDCA) / <0.1 % (*t*-THCA/GlcA-tetrol) percentile range, which indicates that carriers are not likely to be identified with either UPLC-MS/MS or FIA-MS/MS when combining both ratios. Unfortunately, we did not have DBS of CTX newborns. In our previous work where we described similar FIA-MS/MS [4] and UPLC-MS/MS [5] methods, CTX newborn DBS were available, and when we plotted these results in the graphs of our current study (Fig. 1), these clearly fell in the CTX range. This suggests that DBS of CTX newborns can be identified solely by using the combination of the GlcA-tetrol/*t*-CDCA ratios and *t*-THCA/GlcA-tetrol ratios with either UPLC-MS/MS or FIA-MS/MS. Quantification of GlcA-tetrol in combination with the GlcA-tetrol/*t*-CDCA ratio yields an even better separation between newborn DBS and DBS from untreated CTX patients (compare Fig. 1a with 1b, and 1c with 1d) and could be used as first tier screening method or as a second tier after an abnormal first tier. The choice between FIA-MS/MS or UPLC-MS/MS, however, depends on the MS/MS platform already available in the NBS laboratory, and this may change in the future. If more diseases are added that require UPLC-MS/MS in the negative ion mode, such as metachromatic leukodystrophy [9], a first tier UPLC-MS/MS including CTX ratio screening could be an option. As many screening laboratories do not use first tier UPLC-MS/MS -certainly not in the negative ion mode-, the FIA-MS/MS method presented here represents a viable option to start CTX newborn screening but will necessitate polarity switching and thus will extend the analyses time per sample.

## Supplementary Material

sm1

sm2

sm4

sm5

sm3

## Data Availability

Data will be made available on request.

## Footnotes

1 CTX, Cerebrotendinous Xanthomatosis; GlcA-tetrol, cholestanetetrol glucuronide; *t*-THCA, tauro-trihydroxycholestanoic acid; NBS, newborn screening; *t*-CDCA, tauro-chenodeoxycholic acid; FIA, flow injection analysis; DBS, dried blood spot.
