# Supplementary material for: Newborn screening for Cerebrotendinous Xanthomatosis: A retrospective biomarker study using both flow-injection and UPLC-MS/MS analysis in 20,000 newborns
Source: Clin Chim Acta. Author manuscript; Available in PMC 2023 Jul 31. (PMC10387442; doi:10.1016/j.cca.2022.12.011)
Supplement: sm1 [file NIHMS1917525-supplement-sm1.docx]

**Supplementary material 1: FIA-MS/MS and UPLC-MS/MS method**

*FIA-MS/MS system and setup*

The LC system consisted of an LX-50 autosampler and pump and a QSight 220 CR mass spectrometer (PerkinElmer, Finland). Seven microliter of sample was injected on the mass spectrometer in a 20 µl loop (Partial Loopfill). Elution was isocratic at 100% Neo MSMS Flow Solvent (PerkinElmer, Finland). The flow-profile was: 0.00-0.20 min; 0.2 ml/min, 0.20-0.21 min; 0.2 to 1.0 ml/min, 0.22-0.50 min; constant flow of 1.0 ml/min, 0.50-0.51: 1.0 to 0.2 ml/min, 0.51-0.60 min: constant flow of 0.2 ml/min. Including equilibration and injection run to run time was 1 min and 5 seconds.

*FIA-MS/MS pump settings*


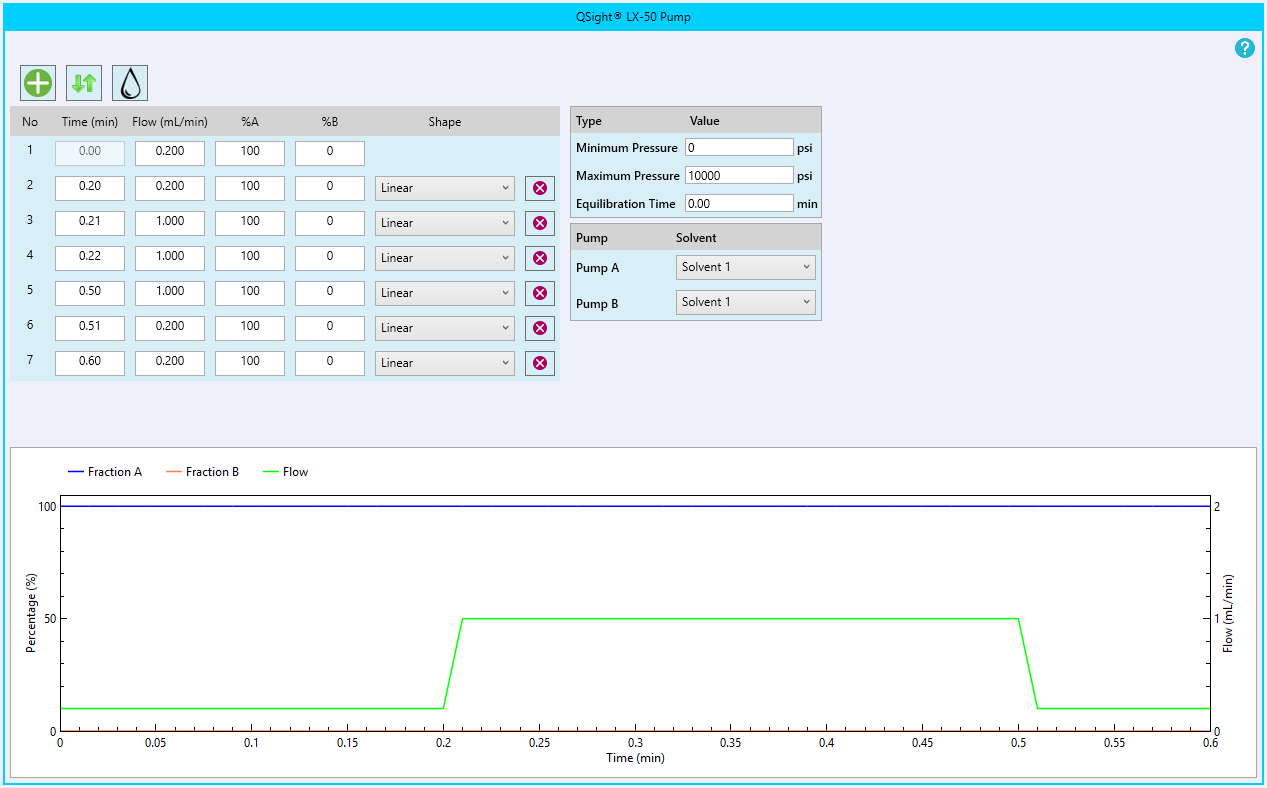


*UPLC-MS/MS system and setup*

The LC system consisted of a LX-50 autosampler, pump and column oven (set at 40°C), an Acquity UPLC HSS T3 1,8 µm VanGuard pre-column, 2.1 x 5 mm and as analytical column the Acquity UPLC HSS T3 1.8 µm 2.1 x 50 mm coupled to a QSight 220 CR mass spectrometer. Seven microliter of sample was injected on the mass spectrometer in a 20 µl loop (Partial Loopfill). Flow was kept at 0.5 ml/min, all gradient steps were linear. Eluant A consisted of ultrapure water:acetonitrile 1:1 (v/v) with 0.1% (v/v) formic acid. Eluant B consisted of isopropanol:acetonitrile 4:1 (v/v) with 0.1% (v/v) formic acid. The elution profile was: 0.00-1.10 min; isocratic 30% eluant B, 1.10-1.30 min; 30% to 100% eluant B, 1.30-1.31 min; 100% to 30% eluant B, 1.32-1.50; isocratic 30% eluant B. Including equilibration and injection run to run time was 2 min and 6 seconds.

*UPLC-MS/MS pump settings*


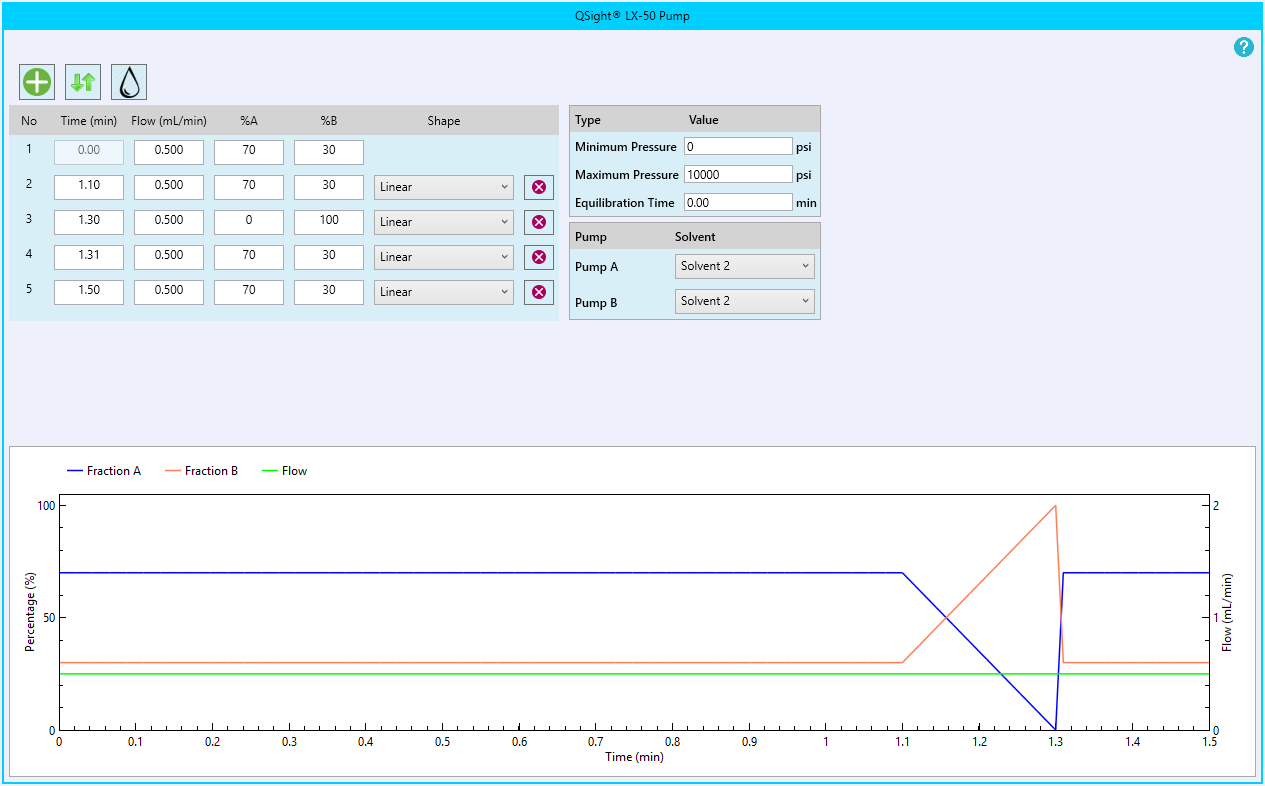


*FIA-MS/MS and UPLC-MS/MS autosampler settings*


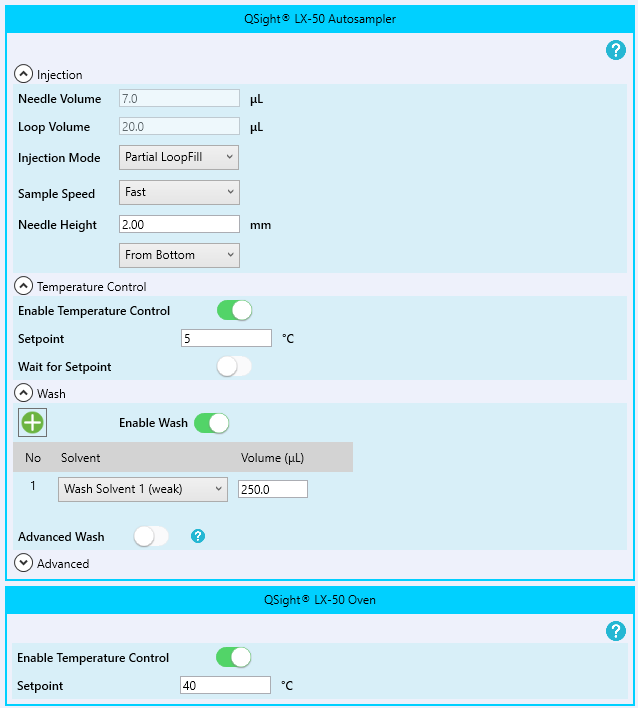


*Mass spectrometric settings FIA- and UPLC-MS/MS*

| **Analyte** | **Q1 m/z** | **Q3 m/z** | **Entrance voltage (V)** | **Collision cell lens (eV)** | **Collision energy (eV)** |
| --- | --- | --- | --- | --- | --- |
| t-CDCA | 498.3 | 79.9 | -110 | 135 | 82 |
| t-THCA | 556.3 | 79.9 | -120 | 150 | 94 |
| g-CDCA | 448.3 | 74.0 | -76 | 168 | 45 |
| GlcA-tetrol | 611.4 | 85.0 | -100 | 150 | 61 |
| GlcA-tetrol-d6 | 617.4 | 85.0 | -100 | 150 | 61 |

| **Parameter** | **FIA-MS/MS** | **UPLC-MS/MS** |
| --- | --- | --- |
| Drying gas (psi) | 100 | 100 |
| HSID temperature (°C) | 270 | 270 |
| Nebulizer gas 1 (psi) | 150 | 150 |
| ElectroSpray V1 Neg (V) | -5850 | -5850 |
| Source temperature (°C) | 400 | 500 |

Needle wash: acetonitril:ultrapure water 7:3 v/v

Seal wash: ultrapure water:methanol 9:1 v/v
