# Supplementary material for: Newborn screening for Cerebrotendinous Xanthomatosis: A retrospective biomarker study using both flow-injection and UPLC-MS/MS analysis in 20,000 newborns
Source: Clin Chim Acta. Author manuscript; Available in PMC 2023 Jul 31. (PMC10387442; doi:10.1016/j.cca.2022.12.011)
Supplement: sm2 [file NIHMS1917525-supplement-sm2.docx]

**Supplementary material 2: GlcA-tetrol/t-CDCA and t-THCA/GlcA-tetrol ratios and the concentrations GlcA-tetrol, t-THCA, t-CDCA concentrations**

**
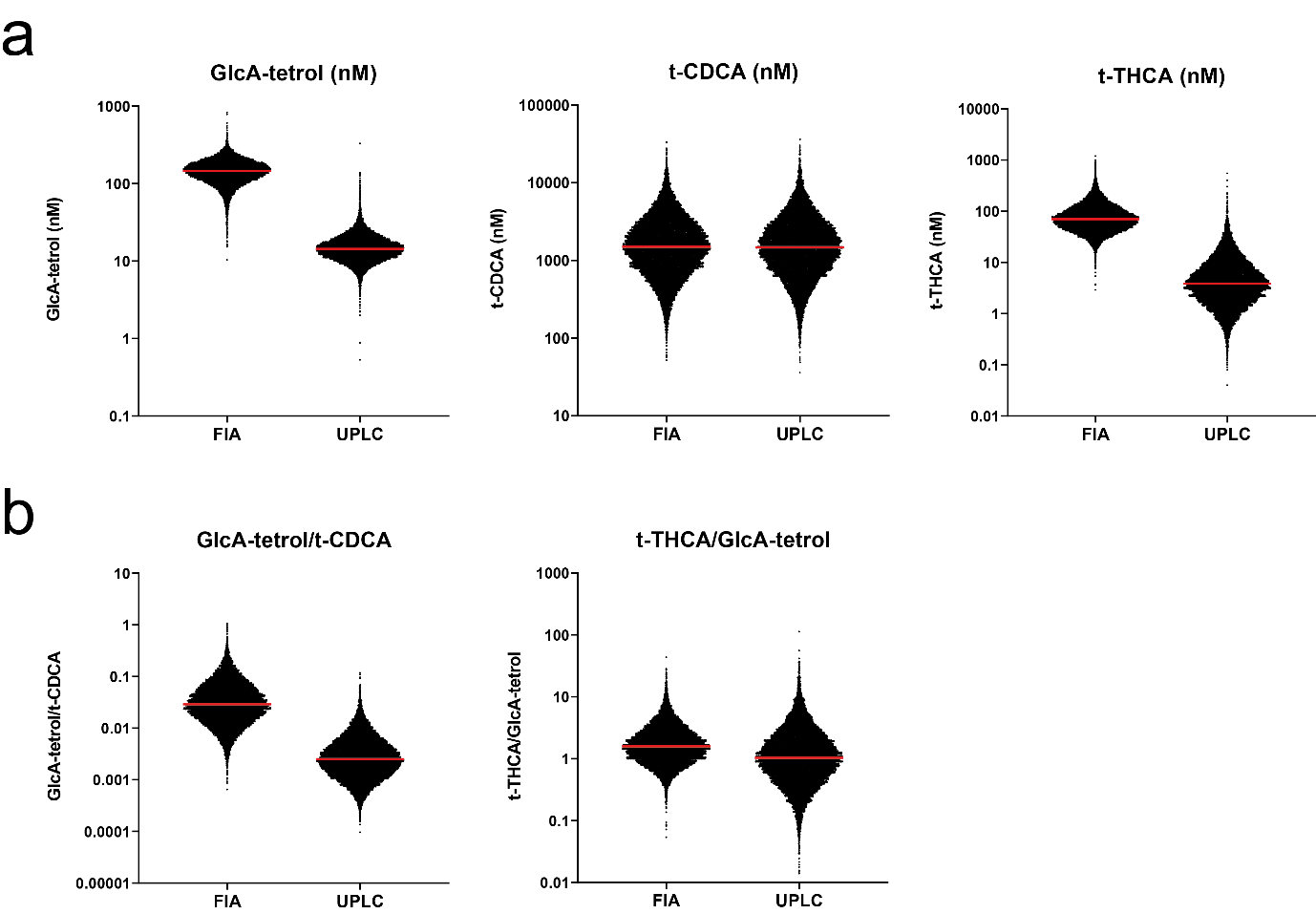
**

c


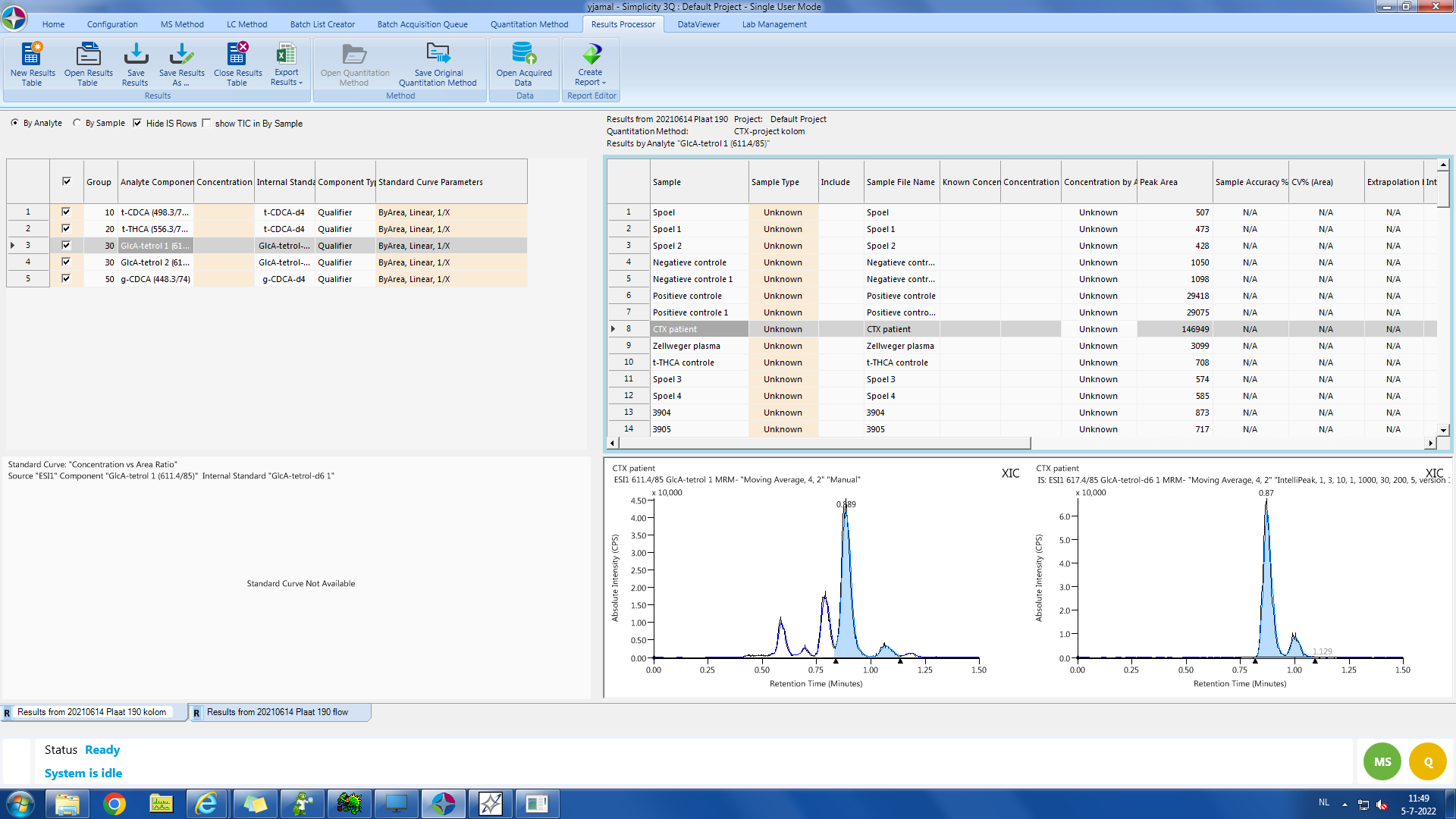


GlcA-tetrol

^2^H_6_-GlcA-tetrol

Overview of metabolite concentrations and ratios (A) Concentrations of GlcA-tetrol, t-CDCA and t-THCA in 20,076 newborns plotted on a ^10^log-scale, red line is the median. (B) GlcA-tetrol/t-CDCA and t-THCA/GlcA-tetrol ratios in 20,076 newborns as calculated from peak areas. (C) Extracted ion chromatogram of GlcA-tetrol (left) of CTX1 and the ^2^H_6_-GlcA-tetrol internal standard (right), blue indicates the integrated area. Despite the use of a stable isotope-labeled internal standard for GlcA-tetrol, the apparent GlcA-tetrol concentrations in newborns using the FIA-MS/MS method were about ten-fold higher than those measured with UPLC-MS/MS (figure S2A). This is most likely due to the higher background levels for FIA detection of GlcA-tetrol. Generally, only background is detected in controls, even using UPLC detection the GlcA-tetrol peak is negligible/noise. In CTX DBS where the GlcA-tetrol levels are much higher, GlcA-tetrol concentrations were only around 2-fold higher in FIA-MS/MS when compared to UPLC-MS/MS. As can be seen in S2C, other isomers of GlcA-tetrol that were present in the CTX samples (more accurately: peaks in the extracted ion chromatogram of GlcA-tetrol not present in the ^2^H_6_-GlcA-tetrol internal standard) but were not integrated as GlcA-tetrol in the UPLC-MS/MS did contribute to the FIA-MS/MS signal. As t-CDCA concentrations were similar in both methods, the apparently ten-fold higher GlcA-tetrol concentrations in FIA-MS/MS also resulted in a ten-fold higher value of the GlcA-tetrol/t-CDCA ratio for FIA-MS/MS. Similarly, apparent t-THCA concentrations (calculated on the ^2^H_4_-t-CDCA internal standard) were about ten-fold higher in FIA-MS/MS when compared to UPLC-MS/MS. This also is likely due to the low concentrations in control DBS and the fact that ^2^H_4_-t-CDCA was used as internal standard that likely does not correct well for interferences. Consequently, the t-THCA/GlcA-tetrol ratios were comparable between both methods as the respective t-THCA and GlcA-tetrol concentrations cancelled out the ten-fold difference in non CTX newborns.
