# Supplementary material for: Newborn screening for Cerebrotendinous Xanthomatosis: A retrospective biomarker study using both flow-injection and UPLC-MS/MS analysis in 20,000 newborns
Source: Clin Chim Acta. Author manuscript; Available in PMC 2023 Jul 31. (PMC10387442; doi:10.1016/j.cca.2022.12.011)
Supplement: sm4 [file NIHMS1917525-supplement-sm4.docx]

**Supplementary material 4:**

**Precision of CTX1, CTX2, and negative control DBS for FIA- and UPLC-MS/MS**

| **FIA-MS/MS** | GlcA-Tetrol  (nM) | t-CDCA  (nM) | t-THCA  (nM) | GlcA-tetrol/t-CDCA | t-THCA/GlcA-tetrol |
| --- | --- | --- | --- | --- | --- |
|  |  |  |  |  |  |
| **CTX1 (n=160)** |  |  |  |  |  |
| Mean | 1921 | 594 | 52.7 | 1.016 | 0.089 |
| SD | 255 | 87 | 14.3 | 0.140 | 0.024 |
| CV% | 13.3% | 14.7% | 27.0% | 13.8% | 27.1% |
|  |  |  |  |  |  |
| **CTX2 (n=78)** |  |  |  |  |  |
| Mean | 4525 | 140 | 54.7 | 8.85 | 0.046 |
| SD | 385 | 24 | 10.4 | 1.57 | 0.009 |
| CV% | 8.5% | 16.9% | 18.9% | 17.7% | 19.7% |
|  |  |  |  |  |  |
| **Neg. control**  **(n=237)** |  |  |  |  |  |
| Mean | 135 | 367 | 45.0 | 0.1135 | 1.231 |
| SD | 42 | 43 | 12.0 | 0.040 | 0.5684 |
| CV% | 31.3% | 23.4% | 26.7% | 35.1% | 46.2% |

| **UPLC-MS/MS** | GlcA-Tetrol  (nM) | t-CDCA  (nM) | t-THCA  (nM) | GlcA-tetrol/t-CDCA | t-THCA/GlcA-tetrol |
| --- | --- | --- | --- | --- | --- |
|  |  |  |  |  |  |
| **CTX1 (n=160)** |  |  |  |  |  |
| Mean | 885 | 536 | 7.31 | 0.641 | 0.0214 |
| SD | 78 | 62 | 2.11 | 0.105 | 0.0053 |
| CV% | 8.8% | 11.6% | 28.8% | 16.4% | 24.6% |
|  |  |  |  |  |  |
| **CTX2 (n=78)** |  |  |  |  |  |
| Mean | 2394 | 83.4 | 10.9 | 10.2 | 0.013 |
| SD | 172 | 6.9 | 2.4 | 1.7 | 0.003 |
| CV% | 7.2% | 33.3% | 22.0% | 16.6% | 21.9% |
|  |  |  |  |  |  |
| **Neg. control**  **(n=237)** |  |  |  |  |  |
| Mean | 12.8 | 275 | 11.3 | 0.0153 | 3.16 |
| SD | 3.6 | 45 | 3.17 | 0.0060 | 1.99 |
| CV% | 28.1% | 16.5% | 16.5% | 39.5% | 62.8% |
